# Tailored optical propulsion forces for controlled transport of resonant gold nanoparticles and associated thermal convective fluid flows

**DOI:** 10.1038/s41377-020-00417-1

**Published:** 2020-10-27

**Authors:** José A. Rodrigo, Mercedes Angulo, Tatiana Alieva

**Affiliations:** Facultad de Ciencias Físicas, Ciudad Universitaria s/n, Universidad Complutense de Madrid, 28040 Madrid, Spain

**Keywords:** Nanophotonics and plasmonics, Optical manipulation and tweezers, Laser material processing

## Abstract

Noble metal nanoparticles illuminated at their plasmonic resonance wavelength turn into heat nanosources. This phenomenon has prompted the development of numerous applications in science and technology. Simultaneous optical manipulation of such resonant nanoparticles could certainly extend the functionality and potential applications of optothermal tools. In this article, we experimentally demonstrate optical transport of single and multiple resonant nanoparticles (colloidal gold spheres of radius 200 nm) directed by tailored transverse phase-gradient forces propelling them around a 2D optical trap. We show how the phase-gradient force can be designed to efficiently change the speed of the nanoparticles. We have found that multiple hot nanoparticles assemble in the form of a quasi-stable group whose motion around the laser trap is also controlled by such optical propulsion forces. This assembly experiences a significant increase in the local temperature, which creates an optothermal convective fluid flow dragging tracer particles into the assembly. Thus, the created assembly is a moving heat source controlled by the propulsion force, enabling indirect control of fluid flows as a micro-optofluidic tool. The existence of these flows, probably caused by the temperature-induced Marangoni effect at the liquid water/superheated water interface, is confirmed by tracking free tracer particles migrating towards the assembly. We propose a straightforward method to control the assembly size, and therefore its temperature, by using a nonuniform optical propelling force that induces the splitting or merging of the group of nanoparticles. We envision further development of microscale optofluidic tools based on these achievements.

## Optofluidics: Light forces for controlling heat nanosources and fluid flows

New opportunities for controlling the motion of laser-heated gold nanoparticles and the associated convection currents in the surrounding fluid are achieved by using tailored light forces. The procedure has been developed by Jose Rodrigo and colleagues at the Complutense University of Madrid. It is based on illuminating nanospheres at wavelengths that create resonant oscillations in surface electrons, a phenomenon known as plasmon resonance. This allows sufficient absorbance of light for the nanospheres to become a heat source. The tailored light forces gave fine control over the direction and speed of the hot nanospheres, both individually and in groups. The refined ability to form, merge and split clusters of nanoparticles could offer new applications in optofluidics—the science of controlling fluids and materials they contain with light.

## Introduction

Light-induced manipulation of micro- and nanosize objects is an active research field with numerous applications in science and technology^1,2^. Well-known laser tweezers, based on intensity-gradient confinement optical forces enabling position control of micro- and nanoparticles (NPs), have played a dominant role as optical manipulation tools in cell biology, material assembly, light-matter interaction physics and chemistry^3–5^. The development of optical tweezers created by structured laser beams has expanded the functionality of optical manipulation tools. The combined application of intensity-gradient confinement forces (trapping forces) and phase-gradient propulsion forces (scattering forces) has prompted the exploration of more sophisticated ways to control the motion and collective behaviour of particles^1,6–8^. For example, the assembly and disassembly of silver NP (150 nm diameter) lattices have been explored by using an optical line trap with a tuneable transverse phase-gradient force^7^. Ordered assemblies of particles linked by electrodynamic interparticle forces in an optical field, known as optical matter, have been created by the intensity/phase-gradient forces and electrodynamic binding forces^9^ that particles experience in the laser trap. Phase-gradient forces have been applied to determine the structure and stability of optical matter arrays of metal NPs^7^. An optical vortex ring trap with a uniform phase-gradient force has been used for rotation of multiple dielectric microparticles and metal NPs against a substrate^10–14^. Its application as a micropump device has been suggested^10^.

Tuneable transverse phase-gradient forces have been applied for programmable light-driven transport of dielectric microparticles along arbitrary three-dimensional (3D) trajectories by using the so-called freestyle laser trap^8,15^. The freestyle laser trap allows straightforward creation of the optical transport route^15^ with independent control of the phase-gradient propulsion force tailored along it^16^. Robotic-like optical transport of metal NPs driven by phase-gradient forces along a 2D curve, whose shape can be easily changed according to the considered transport operation, has also been achieved by using a freestyle laser trap^17^.

Optical manipulation of metal NPs has attracted special interest in the last decade because of their size- and shape-dependent as well as wavelength-tuneable optothermal properties. In particular, illumination of a metal NP or nanostructure with a wavelength close to the plasmon resonance transforms it into an efficient local heat source due to the enhanced light absorption. A resonant metal NP can easily reach a temperature that can alter the physical environment, for example, the viscosity of the fluid surrounding it^18,19^. This behaviour is the basis of numerous applications, such as photothermal therapy, drug delivery, photothermal and photoacoustic imaging and thermal optofluidics^18–28^. Thermal optofluidics relies on the optothermal control of fluid motion, which can be achieved by using resonant plasmonic structures. Different physical phenomena are involved in the generation of such fluid flows^18,19,21,29^. A deeper understanding of the rather complex thermal mechanisms providing fluid motion at the microscale would allow control of the direction, velocity and extension of the fluid flows and, in turn, of the optothermal transport of colloidal particles. It has been shown that an increase in the local temperature of a metal NP (or an NP assembly) is responsible for the appearance of convective fluid flow. Its configuration depends, in particular, on the position of the NP (usually attached on a floor or ceiling substrate) in the chamber and the width of the latter^19,21,29^. By illuminating periodic arrays of closely spaced plasmonic nanostructures (lithographically fixed on a floor glass substrate), the collective heating produces a short-range fluid convection motion with a speed of ∼1 µm/s, which can be increased up to ∼10 µm/s by using an optically absorptive substrate^30^. The photothermal-induced natural convection flow has also been applied for the assembly and deposition of nanoparticles onto surfaces^20^. One of the parameters playing an important role in the fluid dynamics is the temperature of the local heater. A significant local increase in the temperature, in the range of Δ*T* ∼ 70 −250 K, produces the temperature-induced Marangoni effect at the liquid water/superheated water interface due to nanobubble formation, which allows a fluid flow speed of 15–30 µm/s to be reached by optically heating a single gold sphere with a diameter of 100 nm^29^. For temperatures above the microbubble formation threshold (*T* ∼ 580 ± 20 K)^31,32^, the so-called temperature-induced Marangoni effect^33^ provides strong long-range convection flow. This regime has been applied as a lithographic tool for controlled deposition of plasmonic NPs onto the same plasmonic substrate (a film of gold nano-islands fixed onto a glass wafer) required for microbubble generation^34^. Other optofluidic mechanisms, such as thermophoresis or the Seebeck effect with promising technological applications^19^, are outside the scope of the presented experimental study.

This work pursues two goals. The first one is to demonstrate stable optical transport of single and multiple gold NPs (spheres of radius 200 nm) whose speed is controlled by an easily tailored phase-gradient propulsion force. While stable optical trapping of metal NPs has been demonstrated before^4,5,35,36^, here, for the first time to our knowledge, the resonant laser wavelength is used for programmable particle motion. The high light absorption of the resonant NP converts it into a heat source. Our second goal is to study the formation and manipulation of a moving heat source that is able to induce significant convective fluid flows. Previous works have mostly focused on optofluidic effects created by fixed plasmonic structures. Here, we report the first evidence of fluid flow originating from a heat source moving with a controlled speed along an optical trap. The spatial and temporal control of the optical propulsion force allows changing of the fluid streams as well as dividing/merging of the heat source. The combination of the optical heating of NPs with their simultaneous programmable transport along the desired trajectory thus breaks ground for the creation of more versatile optofluidics tools.

The article is organized as follows. The next section starts with an experimental study of the dynamics induced by the tailored propulsion forces exerted over a single resonant gold NP immersed in water. The results for traps with different propulsion force designs are compared with numerical simulations. Then, we consider the movement of multiple NPs in the same traps. The resonant NPs heated in the optical trap self-organize into a group, which behaves as a persistent optothermal convertor inducing fluid flow towards it in the trap plane. In the last subsection, a technique for dividing and merging the groups of NPs (acting as heating sources) within the trap is proposed. Section 3 is devoted to a discussion of our findings and concluding remarks. The principles of the optical propulsion force design, the simulation method used to predict the NP transport along a targeted trajectory and the experimental setup are briefly explained in Section 4 (more details can be found in the Supplementary Information).

## Results

To study the dynamics of resonant gold NPs with a radius of 200 nm, we used a freestyle trap^16^ created by a circularly polarized polymorphic beam of wavelength *λ*_0_ = 532 nm. This kind of beam can be designed to be focused into a laser curve with an arbitrary shape and the desired intensity and phase gradient distributions along it. For simplicity, in this case, we have chosen a ring trap of radius *R* = 4 µm with uniform intensity. Therefore, the phase gradient distribution is the only origin of the optical force propelling the NPs along the curve. The optical propulsion force *F*_*φ*_ ∝ *I*(*R*)*ξ*(*φ*), at a point expressed in polar coordinates (*R*, *φ*), is proportional to the product of the intensity *I*(*R*) and phase gradient *ξ*(*φ*) = *Ψ*′(*φ*)/*Rk*_0_, where *Ψ*′ (*φ*) is an azimuthal derivative of the electric field phase and *k*_0_ = 2*π*/λ_0_; see Eq. (3) in Section 4 (Methods) and the Supplementary Information. Then, a linear increase in the tangential speed *v*_*φ*_ = *R*d*φ*/d*t* is expected as the strength of the phase gradient *ξ*(*φ*) increases. This tendency has been confirmed in previous works by using optical vortex traps of different topological charges *m* and thus with constant *ξ*(*φ*) ∝ *m*; see, for example, ref. ^6^. The laser trap was created in proximity (∼200 nm) to the glass cover slip to provide stable axial confinement of the resonant NPs.

### Transport of a resonant gold NP guided by a tailored optical propulsion force

**Fig. 1:**
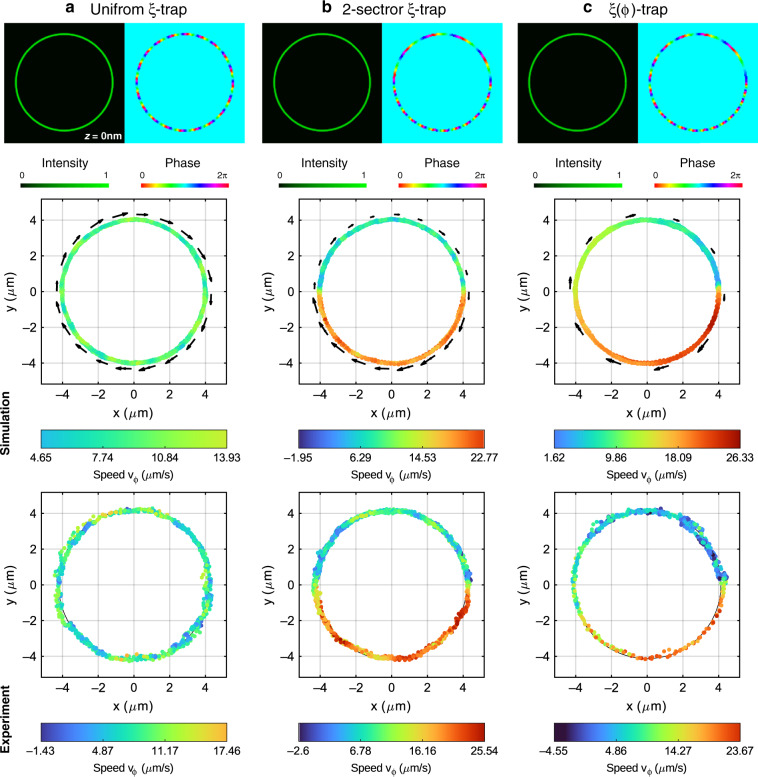
Predicted and measured trajectories of an optically transported NP (gold sphere of radius 200 nm) around different optical ring traps. **a** Results for the case of the ring uniform *ξ*-trap, **b** ring 2-sector *ξ*_1,2_-trap and **c** ring *ξ*(*φ*)-trap. The intensity and phase distribution of the trapping beam (optical ring trap) are displayed in the first row for the uniform *ξ*-trap (**a**), 2-sector *ξ*_1,2_-trap (**b**) and *ξ*(*φ*)-trap (**c**). These distributions have been used in the numerical simulation, providing the predicted trajectory and tangential speed *v*_*φ*_ of the NP (gold sphere of radius 200nm) for each optical trap, as displayed in the second row. The numerical simulation results are in good agreement with the experimental results (measured NP position, tracking duration of 20s) shown in the last row; see Videos S1–S3 for each corresponding case

In this study, we considered the optical propulsion forces created by three different types of phase-gradient profiles *ξ*(*φ*). The corresponding phase *Ψ*(*φ*) prescribed along the ring trap is shown in the first row of Fig. 1. The first phase profile, see Fig. 1(a), is uniform, yielding a phase gradient strength *ξ*(*φ*) ≡ *ξ*_*u*_ = 0.07 in the whole ring trap, which will be referred to as the uniform *ξ*-trap. The second type of ring trap was designed to exert different constant propulsion forces in two sectors. Specifically, this 2-sector *ξ*_1,2_-trap comprises a first sector *φ*_1_ ∈ [0, *π*) where the strength of the phase gradient is *ξ*_1_ = 0.04 and a second sector *φ*_2_ ∈ [*π*, 2*π*) with *ξ*_2_ = 0.1; see Fig. 1b. A more sophisticated phase profile can be achieved by tailoring a nonuniform phase gradient along the transport trajectory in the framework of the freestyle trap^16^. Thus, in the third case, we have applied a linearly increasing phase gradient strength *ξ*(*φ*) ∝ *φ* with *ξ* ∈ [0, 0.13], which is further referred to as the *ξ*(*φ*)-trap; see the corresponding phase profile in Fig. 1c. Since *m* = −20, the NP will undergo a clockwise rotation around the ring trap.

Let us first study the motion of the gold NP in the uniform *ξ*-trap when a constant optical propulsion force was exerted over the NP (see Video S1). The light power at the input aperture of the objective lens was *P* = 40 mW, corresponding to the irradiance *I* = 0.54 mW/μm^2^ in this ring *ξ*-trap. The tracking of the NP position, see the third row of Fig. 1a, shows that the NP travels around the ring with a mean speed of <*v*_*φ*_> = 7 µm/s with a standard deviation of 3 μm/s. The measured radial stiffness of the ring trap is 1.3 pN/μm.

From the speed histograms, we inferred that the origin of the observed random fluctuations of the NP position in both the tangential and radial directions is the Brownian thermal noise, which indeed follows a normal distribution. To confirm this fact, we performed a numerical simulation of the NP dynamics taking into account such random position fluctuations; see the Supplementary Information. Indeed, the numerical simulation predicted that the NP travels around the ring trap with a mean speed of <*v*_*φ*_> =9.4 μm/s with a standard deviation of 3 μm/s; see Fig. 1a. Therefore, the experimental results are in reasonably good agreement with the theoretical predictions.

The 2-sector *ξ*_1,2_-trap was designed to exert a constant propulsion force whose magnitude in the second sector is 2.5× that in the first sector. Therefore, the speed of the NP in the second sector should be <*v*_*φ*,2_> = 2.5<*v*_*φ*,1_>. The experimental results displayed in Fig. 1b demonstrate that the speeds of the NP in the first and second sectors are different (see Video S2). Indeed, the mean speeds of the NP in the first and second sectors are <*v*_*φ*,1_> = 7 μm/s and <*v*_*φ*,2_> = 20 μm/s, respectively, both with a standard deviation of 3 μm/s. This result indicates that the NP is 2.86× faster in the second sector than in the first sector, while the corresponding numerical simulation predicted a speed increase of 2.47×, with <*v*_*φ*,1_> = 7.35 μm/s and <*v*_*φ*,2_> = 18 μm/s; see Fig. 1b.

We observe that the NP speed in the first sector coincides with that measured in the uniform *ξ*-trap despite *ξ*_1_ < *ξ*_*u*_. This fact is explained by the difference in the light power applied in the two cases. The estimated irradiance of the optical field in the cases of the 2-sector *ξ*_1,2_-trap and *ξ*(*φ*)-trap is *I* = 0.73 mW/μm^2^. We recall that the propulsion force is proportional to the product of the intensity and phase gradient. Moreover, since the considered gold NP is resonant with the trapping laser wavelength, it experiences a temperature increase, which in turn reduces the viscosity of the water surrounding the NP; see the Supplementary Information. Thus, the gold NP experiences an estimated temperature increase of Δ*T* ~ 31 K in the uniform *ξ*-trap and Δ*T* ~ 40 K in the rest of the experiments (2-sector *ξ*_1,2_-trap and *ξ*(*φ*)-trap). The larger reduction in the water viscosity in the 2-sector *ξ*_1,2_-trap associated with the NP temperature also contributes to the speed increase of the NP. In Fig. 1c, it is demonstrated that the speed of the NP in the *ξ*(*φ*)-trap (see Video S3) almost linearly increases as a function of the polar angle, *v*_*φ*_(*φ*) ∝ *φ*, as the corresponding numerical simulation predicted.

**Fig. 2:**
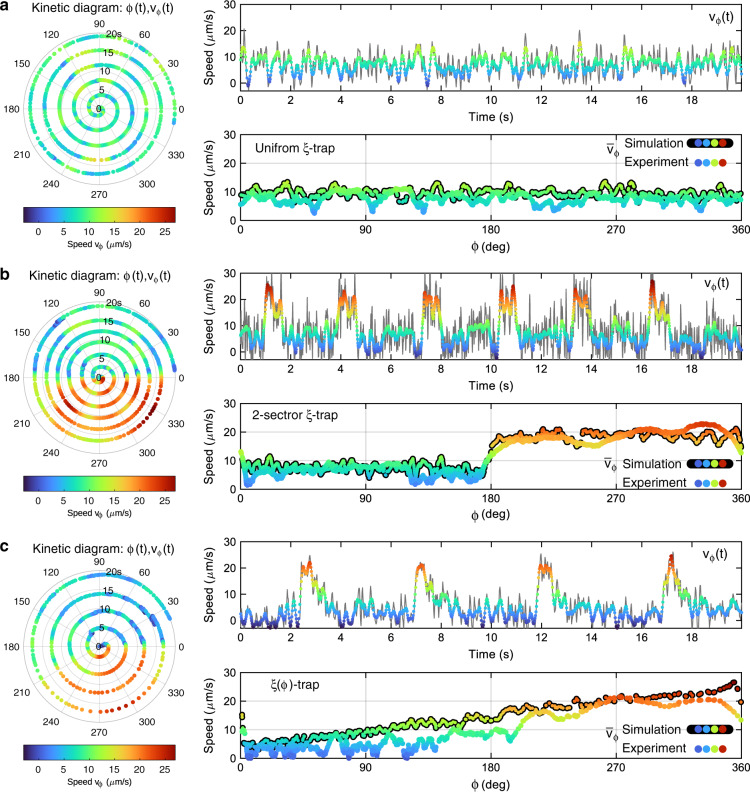
Dynamics of the light-driven transport of the NP. **a** Kinetic diagram and speed profiles of the NP confined in the ring uniform *ξ*-trap, **b** ring 2-sector *ξ*_1,2_-trap and **c** ring *ξ*(*φ*)-trap. These experimental results correspond to the temporal evolution of the NP motion directed by the uniform *ξ*-trap (**a**), 2-sector *ξ*_1,2_-trap (**b**) and *ξ*(*φ*)-trap (**c**). Specifically, the kinetic diagram presents the modulus of the instantaneous tangential speed *v*_*φ*_(*t*) of the NP at angular position *φ*(*t*). The speed *v*_*φ*_(*t*) is also plotted as a function of time, where the grey and coloured lines correspond to the raw and noise-filtered (Savitzky-Golay filter) speed data, respectively. The average tangential speed $\bar v_\varphi (\varphi )$ (experimental) is presented, along with the corresponding numerical simulation data (predicted speed $\bar v_\varphi (\varphi )$), as a function of the angular position *φ* in the ring trap

The temporal evolution of the NP motion was further analysed using the tools displayed in Fig. 2 for the three considered types of optical traps. The first column shows a kinetic diagram presented in polar coordinates, where the radial coordinate corresponds to time (*t*) and the polar angle corresponds to the angular position *φ*(*t*) of the NP. The coloured scale indicates the value of the instantaneous tangential speed *v*_*φ*_(*t*). The temporal variation of the speed, *v*_*φ*_(*t*), is plotted in the second column of Fig. 2. The kinetic diagram for the uniform *ξ*-trap, Fig. 2a, confirms that the NP speed is almost constant except for some eventual speed fluctuations (see also the *v*_*φ*_(*t*) plot) mostly caused by the random thermal force. To compare the experimental and simulation results, we have also presented the plot of speed $\bar v_\varphi (\varphi )$ obtained by time averaging (for each *φ*), which washes out the random fluctuations caused by the thermal noise. The speed profiles $\bar v_\varphi (\varphi )$ of the experimental and numerical simulation results are in good agreement, confirming the expected behaviour of the NP in the uniform *ξ*-trap. The kinetic diagram for the 2-sector *ξ*_1,2_-trap, Fig. 2b, also proves that the optical transport of the NP can be controlled along the trajectory following the prescribed two-level phase-gradient profile. Indeed, as observed in the $\bar v_\varphi (\varphi )$ plot, the experimentally achieved two-level speed profile well fits the simulation results. Finally, the speed of the NP transported in the *ξ*(*φ*)-trap should be *v*(*φ*) ∝ *φ* according to the prescribed phase gradient (*ξ*(*φ*) ∝ *φ*), a tendency that is observed in the experimental and numerical simulation results displayed in the $\bar v_\varphi (\varphi )$ plot in Fig. 2c.

### Optical transport of multiple resonant NPs and simultaneous thermal optofluidic effects

The previous experimental results demonstrate that phase-gradient forces can be applied to control the speed of a single NP, even if the particle is resonant with the trapping laser wavelength. Let us now study the behaviour of multiple resonant NPs in the same trapping conditions.

**Fig. 3:**
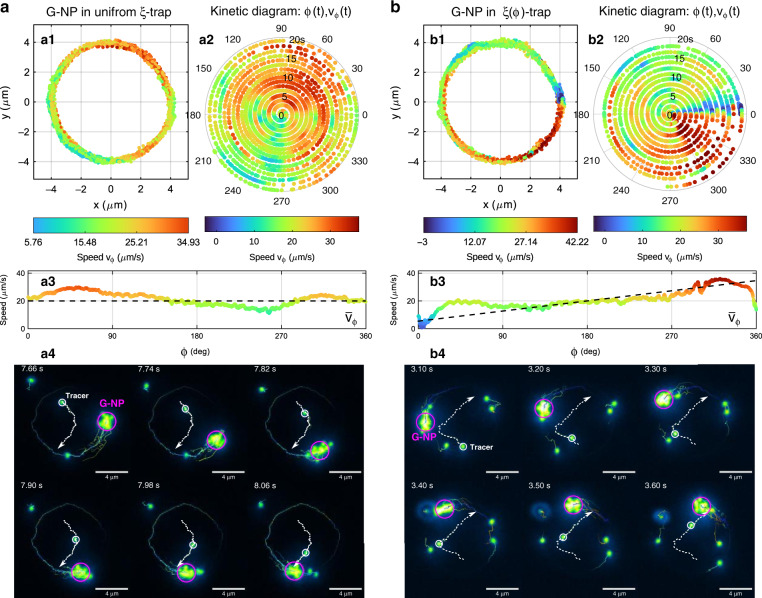
Optical transport dynamics of multiple gold NPs. **a** Measured trajectory, kinetic diagram and speed profile of the hot G-NP optically transported in the uniform *ξ*-trap and **b** in the ring *ξ*(*φ*)-trap. The trajectory of the hot G-NP during the experiments (duration 20s) is displayed in (a1) and (b1), respectively. The corresponding kinetic diagrams are shown in (a2) and (b2). The average speed profile $\bar v_\varphi (\varphi )$ is presented for each case in (a3) and (b3). The experimental darkfield images displayed in the last rows show the G-NP optically transported around the uniform *ξ*-trap (a4) and *ξ(φ*)-trap (b4); see Videos S4 and S5, respectively. The trajectories of the tracer NPs are indicated by the dashed white line

In particular, we study the transport dynamics of multiple gold NPs confined in the uniform *ξ*-trap and *ξ*(*φ*)-trap; see Fig. 3a, b, respectively. Interestingly, the confined NPs rapidly assemble into a stable group travelling around the ring trap. The structure of this group of NPs (further referred to as G-NP) exhibits a relatively low degree of symmetry, as observed in Fig. 3a4 (Video S4) and 3(b4) (Video S5), where the G-NP has been encircled by a pink circle (2 μm diameter) to indicate its position. The number of particles in this circle oscillates between 8 and 15. However, inside the G-NP, there is a more stable group of *N*~5 NPs located closer to each other, further called the core, in a region of diameter ~1.2 μm. Note that the considered gold NPs are coated with charged surfactants to prevent aggregation; thus, the particles are not attached to each other. The position of the G-NP was tracked during the whole experiment (20 s), as displayed in Fig. 3a1, b1 for each optical trap. From these results, we conclude that the G-NP behaves as a sort of quasi-particle whose motion is directed by the optical propulsion force, as observed in the kinetic diagrams shown in Fig. 3a2, b2. In the case of the uniform *ξ*-trap, the G-NP has a mean speed of <*v*_*φ*_> = 20.4 μm/s, and the average speed profile shown in Fig. 3a3 reveals that the G-NP tends to exhibit the behaviour of a single NP transported in the uniform *ξ*-trap. The measured distribution of speed values well fits a normal distribution with a standard deviation of 6 μm/s. In the case of the *ξ*(*φ*)-trap, the G-NP has a mean speed of <*v*_*φ*_> = 17 μm/s. The average speed profile $\bar v_\varphi (\varphi )$ shown in Fig. 3b3 reveals that the G-NP also tends towards the behaviour of a single NP transported in such an *ξ*(*φ*)-trap. The maximum speed of the G-NP is 35 μm/s and 42 μm/s in the uniform *ξ*-trap and *ξ*(*φ*)-trap, respectively.

**Fig. 4:**
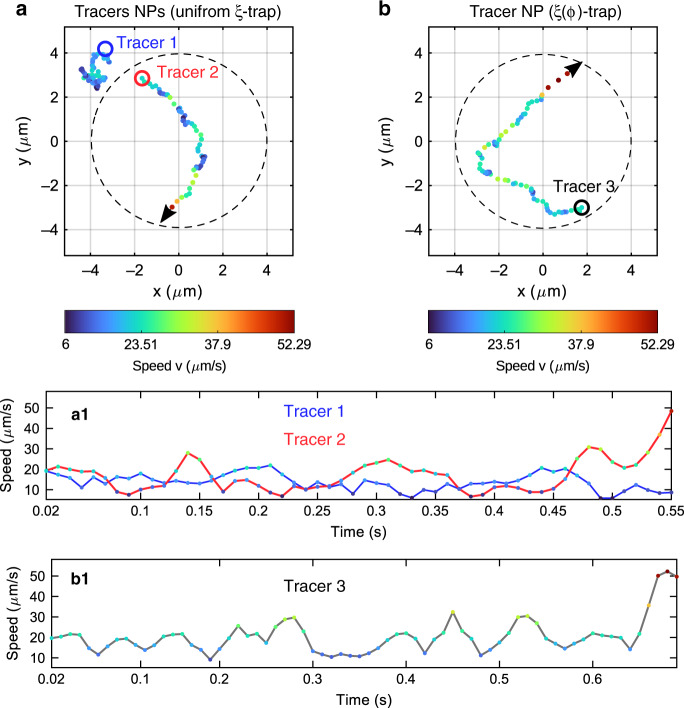
Measured trajectories and speeds of tracer NPs in the presence of a moving heat source (G-NP) driven by a tailored optical propulsion force. **a** Motion of the tracer NPs observed when the hot G-NP is transported in the uniform *ξ*-trap and **b** in the *ξ*(*φ*)-trap. The speed moduli presented as a function of time are displayed in (a1) and (b1), respectively. Tracer 1 experiences Brownian motion since it is too far from the G-NP in the displayed time interval

The G-NP formation produces interesting optothermal effects. Due to light absorption, the temperature of the NPs increases. The temperature increase in the G-NP core is estimated as Δ*T*_GNP_ ~ 200 K, while for a single NP, it is only Δ*T*_NP_ ~ 40 K; see Supplementary Information S3. The hot core of the G-NP warms the surrounding water, reducing its viscosity, which contributes to the speed increase of the G-NP in comparison with the speed of a single NP transported in the trap. Moreover, free (not trapped) gold NPs acting as tracer particles are dragged towards the heat source (G-NP) by the generated convective water flow, as observed in Fig. 3a4, b4 (see also Videos S4 and S5). The action radius of this attractive force in the trap plane is approximately 6 μm. The motion of the tracers is indicated by the dashed white-coloured trajectory in Fig. 3a4, b4. In both cases of the uniform *ξ*-trap and *ξ*(*φ*)-trap, the tracer trajectory instantaneously points towards the heat source as it travels around the ring trap, illustrating the routing of the fluid flow at the microscale. We observe that the trajectories of the tracers located initially at the same distance from the heat source depend on the G-NP speed, which is almost constant in the uniform *ξ*-trap and significantly decreases in the considered region of the *ξ*(*φ*)-trap. Since the G-NP is a moving heat source driven by the phase-gradient propulsion force, the routing of the fluid flow is indirectly controlled by this optical force. The measured position and speed of the tracer NPs are displayed in Fig. 4a, b for each case, the uniform *ξ*-trap and *ξ*(*φ*)-trap, respectively. In the case of the G-NP transported in the uniform *ξ*-trap, we have measured the positions of two tracer NPs, as indicated in Fig. 4a, whose speed moduli are presented as a function of time in Fig. 4a1. In the analysed temporal sequence (*t* = 7.57 − 8.14 s), the first tracer (tracer 1) exhibits Brownian motion, and only the second tracer (tracer 2) is dragged by the flow towards the G-NP. While the modulus of the migration speed of tracer 2 is only slightly higher than the speed of the random motion of tracer 1, it remarkably increases up to *u*_2_~48 μm/s in ~50 ms as the tracer approaches (~2.5 μm) the G-NP. Note that tracer 1 was also dragged towards the G-NP when the distance between them decreased, as observed in the whole time sequence of Video S4. In the case of the G-NP transported in the *ξ*(*φ*)-trap, the measured position and speed of a tracer NP (tracer 3) are displayed in Fig. 4b, b1, corresponding to the time interval *t* = 3.0 − 3.69 s of Video S5. Similar to in the uniform *ξ*-trap case, the migration speed increased remarkably up to *u*_3_ ~ 52 μm/s in ~50 ms next to the G-NP. We underline that the initial positions of the considered tracer NPs are relatively close to the ring trap (~2 μm). However, instead of moving towards the trap, they travel in the direction of the G-NP located far away (~6 μm).

### Optical manipulation of a travelling G-NP heat source

**Fig. 5:**
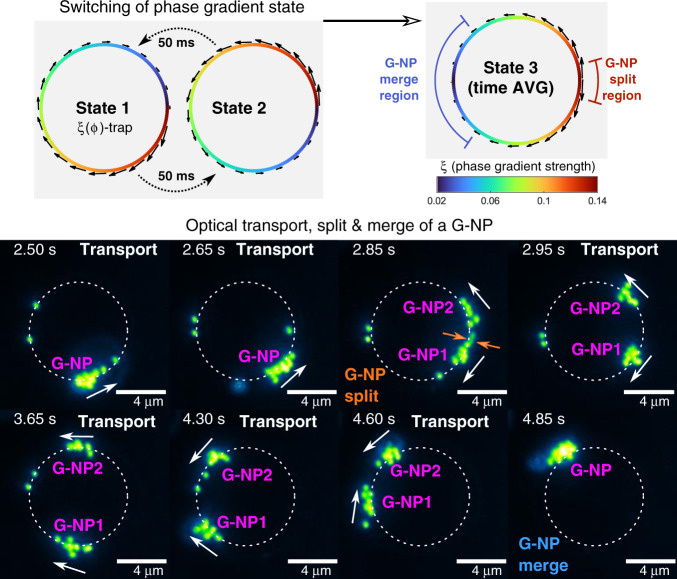
Experimental results illustrating the splitting and merging of a G-NP by switching the phase-gradient state of the ξ(φ)-trap. The switching is performed every 50ms between state 1 (*ξ*(*φ*)) and state 2 (−*ξ*(*φ*−2*π*)), as sketched in the first row. The resulting time-averaged phase-gradient profile (state 3) is responsible for the optical transport, splitting and merging of the G-NPs. Note that the black arrows point in clockwise and anticlockwise directions in the first and second half of the ring trap in state 3, respectively. The second panel shows the achieved manipulation of the G-NP: a single G-NP is transported by state 2 until the switching process is activated. Then, this group of NPs splits into G-NP1 and G-NP2 in the region around *φ* = 0, which then travel in opposite directions towards the merge region, where they finally merge into a joint G-NP again, as observed in Video S6

The development of optofluidics applications based on a moving optothermal source, such as the G-NP studied in the previous section, requires versatile tools for its manipulation, which is illustrated here with a practical example. The previous experiments have demonstrated that the motion of the G-NP can be controlled by the optical propulsion force arising from the phase gradient prescribed along the transport trajectory. In particular, the tailored phase gradient in the *ξ*(*φ*)-trap allows significant slowing down of the G-NP, $\bar v_\varphi (\varphi ) = 0.5$ μm/s in the region *φ* ∈ [0 − 10^○^], followed by a rapid acceleration that increases its speed up to 42 μm/s in a few milliseconds; see Fig. 3b. This rapid speed change of the G-NP inspired us to explore the possibility of G-NP division into two parts by using fast switching of the asymmetric phase gradient state of the *ξ*(*φ*)-trap. Indeed, when the G-NP approaches the low speed region at *φ* ~ 0, a rapid change in the phase gradient state *ξ*(*φ*) to its inverted counterpart −*ξ*(*φ* − 2*π*) propels part of the NPs (belonging to the G-NP) in the opposite direction, as sketched in Fig. 5. These two phase gradient states (further referred to as states 1 and 2) can be continuously interchanged. In the presented experiments, the switching time was 50 ms, which is the limit of the hologram refresh rate of our SLM. This procedure induces the division of the original G-NP into two new ones, referred to as G-NP1 and G-NP2, as observed in Fig. 5 and Video S6. Specifically, due to the asymmetry of phase gradient states 1 and 2, the NPs experience a time-averaged optical propulsion force corresponding to state 3 that directs their motion in the counterclockwise direction in sector *φ* ∈ (0, *π*) and in the clockwise direction in *φ* ∈ (*π*, 2*π*); see Fig. 5. The opposite propulsion forces in the split region *φ* ~ 0 separate the NPs belonging to the original G-NP, thus creating G-NP1 and G-NP2. These two new NP assemblies travel around their corresponding sectors, propelled by a time-averaged propulsion force, and finally merge again into a joint G-NP when approaching the region *φ* ~ *π*. Note that this type of time-averaged propulsion force (state 3) changes along the ring and gradually decreases to zero at *φ*~*π*.

## Discussions

We have experimentally demonstrated stable optical confinement and speed control of a resonant gold NP with a radius of 200 nm in a structured ring trap set in proximity to a ceiling glass substrate. The motion dynamics of the NP observed along the ring are in reasonably good agreement with the expected action of the optical propulsion force originating from the designed transverse phase gradient. Despite the relatively large particle size exceeding the dipole approximation, the propulsion force can still be considered proportional to the product of the irradiance and tangential phase derivative, as follows from the analysis of the observed particle speed in the considered traps. The largest difference between experimental and simulated averaged speeds is observed for the region of weak phase gradient, where the modulus of the directional speed is on the order of the Brownian motion speed. We have shown that a proper phase design allows rapid or slow change of the particle speed without the need for a temporal variation of the trapping beam. Based on these results together with our previous studies, where optical transport of colloidal particles along trajectories of different shapes was demonstrated, we conclude that by utilizing all the advantages of the freestyle trap^8,16,17^, it is also possible to organize efficient transport of resonant metal NPs along arbitrary 2D routes with controlled speed.

We have observed that when multiple NPs are trapped in the structured ring trap, they tend to form a quasi-stable group independent of the configuration of the optical propulsion force. Several particles (~5) located closer to each other take the shape of a core in the group, while others are more susceptible to changes in their position. The movement of the G-NP is similar to the optically directed movement of the single NP. The speed of the G-NP also evidences the tangential phase derivative distribution, but it is almost twice that of the single NP. We attribute this speed increase to the larger extinction cross section of the G-NP and the viscosity change of the surrounding water due to the temperature increase associated with the collective light absorption of the G-NP. The controlled optical transport of the G-NP along a more sophisticated trajectory in the form of a knot circuit with a tailored propulsion force is shown in the Supplementary Information (and Video S7), which again underlines the versatility of the freestyle trap.

Analysis of the movement of free gold NPs (tracers) found in the plane of the optical ring trap reveals their displacement towards the G-NP moving around the ring. We associate these phenomena with convective fluid flows induced by a significant temperature increase in the G-NP. Indeed, the high temperature of the G-NP and therefore of the surrounding medium can produce different optofluidics effects. Taking into account the experimental conditions (trapping near the ceiling substrate) and the estimated temperature of the G-NP (~500 K) and following the reasoning as well as simulation results of a recent work^29^ (where the optothermal flows generated by a single 100 nm gold NP fixed on a ceiling substrate were considered), we suppose that the flow originates from the temperature-induced Marangoni effect at the liquid water/superheated water interface due to nanobubble formation. The speed of the tracer NP and its increase in the proximity of the G-NP, as well as the tracer trajectory always pointing towards the hot G-NP, support our hypothesis. Note that the thermophoretic force cannot explain the observed motion of tracers towards the heat source since its direction in the considered water solution should be from the hot to cold region (positive Soret coefficient)^29^. We have observed that the trajectories followed by the tracers initially located at the same distance from the heat source (~6 μm) have different shapes depending on the constant or variable speed of the G-NP. Therefore, the propulsion force plays a crucial role in the orientation of the heat-induced fluid motion.

We have proposed a technique to control the size of the G-NP by dividing or merging the groups of NPs by fast (50 ms) switching of the traps with a properly designed asymmetric tangential phase gradient. The division and merging process can be recursively applied for controlled creation of multiple G-NPs, which can be used for the generation of more complex fluid flows. For instance, two or more G-NPs can be spatially redistributed by simply transforming the transport route into a reconfigurable network circuit, as reported in ref. ^37^, thereby creating more complex landscapes of travelling optothermal sources enabling micro-optofluidics on demand.

We envision that the use of tailored optical propulsion forces can indeed prompt the development of more versatile thermal optofluidic microsystems based on optical heating and programmable, similar to robotic motion planning, transport of plasmonic NPs.

## Materials and methods

### Design of the optical propulsion forces in a freestyle trap

The circular polarized polymorphic beam associated with the considered ring trap is expressed in Cartesian coordinates (in the input aperture of the objective lens with focal distance f) as the vector

1 $${\mathbf{E}}_0\left( {x,\;y} \right) = {\epsilon}_ \pm \mathop {\int }\nolimits_{\!\!0}^{2\pi } g(\tau ){\mathrm{exp}}\left[ { - \:{\mathrm{i}}\left( {\frac{k}{f}} \right)R\left( {x{\mathrm{cos}}\tau \: + \:y{\mathrm{sin}}\tau } \right)} \right]d\tau$$

where *k* = *k*_0_*n*_*m*_ = 2*πn*_*m*_/*λ*_0_, with *λ*_0_ being the light wavelength and *n*_*m*_ being the refractive index of the surrounding medium, *R* is the radius of the ring trap, and $\epsilon$_±_ = (1,±i) is the circular polarization vector. The complex weight function

2 $$g\left( \tau \right)\: = \:|g\left( \tau \right)|{\mathrm{exp}}\left[ {{\mathrm{i}}\Psi \left( \tau \right)} \right]$$

plays an important role because it allows designing the amplitude (through |*g*(*τ*)|, in the unit of electric field, Vm^−1^) and the phase (through *Ψ*(*τ*)) distributions of the focused beam (**E**, see the Supplementary Information) in the form of the target curve^16^.

We will further consider the case of a uniform intensity distribution along the ring trap that corresponds to constant amplitude |*g*(*τ*)| = *g*_0_, which depends on the light power of the incident beam at the input aperture of the objective lens. Thus, the optical force propelling the particle around the ring trap is described only by the azimuthal component of the scattering force (optical propulsion force). We assume (see the Supplementary Information for further details) that the propulsion force is directly proportional to the phase derivative *Ψ*′(*φ*) and can be written in polar coordinates (*r*, *φ*) in the objective focal plane (the trapping plane) as

3 $$F_\varphi \left( {R,\:\varphi } \right) = \frac{{{\upsigma }}_{{\mathrm{ext}}}}{{Rk_0c}}I\left( R \right)\Psi ^\prime \left( \varphi \right) = \left( {\frac{{\sigma _{{\mathrm{ext}}}}}{c}} \right)I\left( R \right)\xi \left( \varphi \right)$$

where *σ*_ext_ is the extinction cross section of the NP, *I*(*R*) = *n*_*m*_*ε*_0_*c*|**E**(*R*)|^2^/2 is the intensity (irradiance) of the wave incident on the NP immersed in the medium (e.g., water) of refractive index *n*_*m*_, *ε*_0_ is the permittivity of vacuum, and *c* is the light speed in vacuum. Here, we have introduced a dimensionless parameter *ξ*(*φ*) = *Ψ*′(*φ*)/*Rk*_0_ describing the strength of the phase gradient, which is useful for proper comparison between different configurations of the optical propulsion force. Another useful parameter is the global phase accumulation along the entire curve, *m* = *Ψ*(2*π*)/2*π*, which can be considered as a generalization of the beam topological charge for the case of a nonuniform phase distribution along the curve. In the considered experiments, we used a ring trap of radius *R* = 4 μm and *m* = −20 but with different phase-gradient profiles *ξ*(*φ*) prescribed along the ring. The numerical simulation performed to predict the NP optical transport along a targeted trajectory is based on the integration of the 2D Langevin dynamics equation of motion using a splitting-method time-integration scheme (BAOAB)^38–40^; see the Supplementary Information for further details.

### Experimental setup

The experimental setup consists of an inverted darkfield microscope, a programmable reflective SLM (Holoeye PLUTO, pixel size of 8 μm) and a high speed sCMOS camera (Hamamatsu, Orca Flash 4.0, 16-bit grey-level, pixel size of 6.5 μm). To generate the trapping beam, a phase-only hologram addressed into the SLM modulates an input collimated laser beam (Laser Quantum, Ventus, *λ*_0_ = 532 nm, 1.5 W), which is then projected (with a 1× Keplerian telescope) into the input (back) aperture of the microscope objective lens (Olympus UPLSAPO, 1.4 NA, 100×, oil immersion), as reported in^17^.

The gold NPs (Cytodiagnostics, citrate-stabilized spheres with a 200 nm radius immersed in an aqueous solution) were observed under white light illumination (high power LED, SugarCube Ultra) by using an oil immersion darkfield condenser (Nikon, 1.43 NA). The sample was sandwiched between two glass cover slips separated by a spacer ~100 μm thick. Note that the darkfield diaphragm (required for darkfield imaging) was mounted in the imaging system placed outside the microscope (in front of the camera) instead of in the back aperture of the objective lens, thereby preventing cut-off of the projected trapping beam^17^. The darkfield image of the NPs was recorded by a sCMOS camera at 100 frames per second with an exposure time of 1 ms. A notch filter (Semrock, dichroic beamsplitter for 532 nm) redirected the trapping beam into the objective lens, which prevented saturation of the camera by backscattered laser light. In all the considered experiments, position tracking of the NPs was performed for 20 s, which is sufficient because of the high frame rate of the video recording. We underline that the studied optical transport of NPs is stable and was observed over hours in reproducible experiments. The position tracking of the particles was performed by using open source software^41^.

## Supplementary information

Supplementary information for the main text

Single gold nanoparticle in uniform ring trap

Single gold nanoparticle in 2-sector ring trap

Single gold nanoparticle in non-uniform ring trap

GNP in uniform ring trap

GNP in non-uniform ring trap

Split and merge of a GNP by a non-uniform ring trap

Optical transport of nanoparticles and GNP in knot circuit optical trap
