# Supplementary material for: Tailored optical propulsion forces for controlled transport of resonant gold nanoparticles and associated thermal convective fluid flows
Source: Light Sci Appl. 2020 Oct 27;9:181. doi: 10.1038/s41377-020-00417-1 (PMC7589520; doi:10.1038/s41377-020-00417-1)
Supplement: Supplementary file 1 — Supplementary information for the main text [file 41377_2020_417_MOESM1_ESM.pdf]

TAILORED OPTICAL PROPULSION FORCES FOR CONTROLLED TRANSPORT OF RESONANT GOLD NANOPARTICLES AND ASSOCIATED THERMAL CONVECTIVE FLUID FLOWS: SUPPLEMENTARY INFORMATION

José A. Rodrigo,* Mercedes Angulo and Tatiana Alieva

Universidad Complutense de Madrid, Facultad de Ciencias Físicas,

*Ciudad Universitaria s/n, Madrid 28040, Spain**

OPTICAL FORCES ACTING UPON A NP

The optical forces exerted on a colloidal particle is determined by Maxwell's stress tensor and their analysis requires the application of numerical calculation methods. The easily interpreted analytical expression for time-averaged force $\mathbf{F}$, exerted by monochromatic field, is only obtained in the dipole approximation [1–3]

$$\begin{aligned}\mathbf{F} &= \frac{\varepsilon_0\varepsilon_m}{2}\mathrm{Re}\left[\alpha\sum_q E_q\nabla E_q^*\right]\\ &= \frac{\varepsilon_0\varepsilon_m}{4}\alpha'\nabla\left|\mathbf{E}\right|^2 + \frac{\varepsilon_0\varepsilon_m}{2}\alpha''\mathrm{Im}\left(\sum_q \left|E_q\right|^2\nabla\Psi_q\right),\end{aligned} \quad (1)$$

where $\mathbf{E}(\mathbf{r}) = (E_x, E_y, E_z)$ is an electric field vector with $E_q \equiv E_q(\mathbf{r}) = |E_q(\mathbf{r})|\exp[\mathrm{i}\Psi_q(\mathbf{r})]$, ∇ is a gradient operator expressed in the chosen coordinate system and q is a placeholder for the spatial coordinates x, y, z. Here ε_0 is the permittivity of vacuum, $\varepsilon_m = n_m^2$ is the relative permittivity of surrounding medium (usually considered as a real parameter) and $\alpha = \alpha' + \mathrm{i}\alpha''$ is the particle polarizability. The first term in Eq. (1) can be rewritten in the vector form as $\mathbf{F}_\nabla = n_m\alpha'\nabla I/2c$, where $I = n_m\varepsilon_0 c\left|\mathbf{E}\right|^2/2$ is the intensity (irradiance) of the incident wave in the medium and c is the speed of light in vacuum. This conservative force, often called intensity-gradient or dipole force, pulls the particle toward the position of the intensity maximum (minimum) if the dispersive (real) part of polarizability $\alpha' > 0$ ($\alpha' < 0$) and allows for the particle confinement in optical tweezers. The second term describes non-conservative forces arising from light absorption and scattering by the particle and depends on

* jarmar@fis.ucm.es

the dissipative (imaginary) part of the polarizability α''. It is determined by phase gradients of field components and takes into account the field polarization [4, 5]. Since the α'' is always positive then this so called scattering force, $\mathbf{F}_{\rm scat}$, propels the particle in the direction of the phase gradients. In particular, in the case of the freestyle optical trap [6] the axial component of the scattering force has to be compensated by the intensity-gradient force to achieve stable three-dimensional (3D) trap. While the transverse component of the scattering force could help in radial confinement and propels the particle along the designed trajectory (e.g., a ring) in the plane transverse to the beam propagation.

Our goal is to consider the application of such propulsion scattering force for versatile motion control of metal NPs. Strictly speaking the expression Eq. (1) is valid for a Rayleigh particle with radius $a < 0.1\lambda$, with $\lambda = \lambda_0/n_m$ being the light's wavelength in the medium (where the wavevector is $k = k_0 n_m = 2\pi/\lambda$). In this case $\alpha'' = \sigma_{\rm ext}/k = (\sigma_{\rm abs} + \sigma_{\rm scat})/k$, where $\sigma_{\rm ext}$, $\sigma_{\rm abs}$ and $\sigma_{\rm scat}$ are cross sections for extinction, absorption and scattering, correspondingly. As a rule of thumb it is often assumed that the scattering force for a larger particle can also be found as

$$\mathbf{F}_{\rm scat} = \frac{\varepsilon_0 \varepsilon_m}{2k} \sigma_{\rm ext} {\rm Im} \left(\sum_q |E_q|^2 \nabla \Psi_q \right) \quad (2)$$

where $\sigma_{\rm ext}$ depends on the relative permittivity ε_p of the particle, its form, size, etc. and can be calculated using Mie theory [7]. The extinction cross section is $\sigma_{\rm ext} = 0.455\,\mu{\rm m}^2$ for the considered gold NP (radius $a = 200$ nm), at the illumination wavelength $\lambda_0 = 532{\rm nm}$.

In order to study the light-induced motion of resonant metal NPs we have used the so-called freestyle laser trap which allows for both the optical confinement and transport of the particle along arbitrary 3D trajectory with independent control of the optical propulsion force [8, 9]. Notice that the scattering optical force is significantly increased when the incident laser wavelength is near the plasmon resonance of the particle, in which case ${\rm Re}\,[\varepsilon_p] \approx -2\varepsilon_m$ holds. This resonance increases the particle speed along the optically defined trajectory, however, stable 3D trapping is not possible due to strong axial component of the scattering force pushing the particle in the beam's propagation direction, which cannot be compensated by the axial component of the intensity gradient force. Nevertheless, stable 2D confinement and transport of a resonant metal NP is possible against a substrate such as the glass cover-slip. We have applied a freestyle laser trap created by a polymorphic beam [10] which is focused in form of 2D laser curve in the plane transverse to the beam propagation

allowing the optical transport of the particles along the target curve. Note that in the experiment the laser curve can be slightly defocused onto the substrate. In this work, we are interested in the optical control of the particle dynamics along a fixed trajectory which, as an example, is a circumference (ring) of radius R. Here we have used circular polarization that prevents from an anisotropy in the optical forces responsible for persistent NP velocity oscillations when a linear polarized trapping beam is used [11, 12]. Specifically, the circular polarized polymorphic beam associated to the considered ring trap is expressed in Cartesian coordinates (in the input aperture of the objective lens with focal distance f) as the vector

$$\mathbf{E}_0\left(x,y\right)=\epsilon_{\pm}\int_0^{2\pi} g(t)\exp\left[-\mathrm{i}\frac{k}{\mathrm{f}}R\left(x\cos t+y\sin t\right)\right]\mathrm{d}t, \tag{3}$$

where $\epsilon_{\pm}=(1,\pm\mathrm{i})$ is the circular polarization vector. This expression can be rewritten in polar coordinates as a superposition of helical Bessel functions

$$\begin{aligned}\mathbf{E}_0\left(r,\phi\right)&=\epsilon_{\pm}\int_0^{2\pi} g(t)\exp\left[-\mathrm{i}\frac{k}{\mathrm{f}}Rr\cos(t-\phi)\right]\mathrm{d}t\\&=\epsilon_{\pm}\sum_{n=-\infty}^{\infty}(-\mathrm{i})^n\exp\left(-in\phi\right)J_n(\frac{k}{\mathrm{f}}Rr)\int_0^{2\pi} g(t)\exp\left(\mathrm{i}nt\right)\mathrm{d}t.\end{aligned} \tag{4}$$

The complex weight function

$$g\left(t\right)=\left|g\left(t\right)\right|\exp\left[\mathrm{i}\Psi\left(t\right)\right], \tag{5}$$

allows designing the beam's amplitude (through $|g(t)|$, in the unit of electric field, $\mathrm{V\,m^{-1}}$) and phase (through $\Psi(t)$) distributions along the target curve [10].

In paraxial approximation with unlimited objective aperture, the shape of the beam $\mathbf{E}$ in the focal plane is described by a ring of radius R as it is easy to prove by calculating the Fourier transform of $\mathbf{E}_0$ (i.e., $\mathbf{E}=\mathrm{FT}(\mathbf{E}_0)$) [10]. We will further consider the uniform intensity distribution along the ring that corresponds to $|g(t)|=A_0\frac{2\pi R^2}{\lambda \mathrm{f}}$, with A_0 being a constant which depends on the light power of the incident beam at the input aperture of the objective lens. Thus the optical force propelling the particle around the ring trap is described only by the azimuthal component of scattering force, which in considered approximation is directly proportional to the phase derivative $\Psi'(\varphi)$ as it follows:

$$F_{\varphi}(R,\varphi)=\frac{\varepsilon_0\varepsilon_m}{2k}\sigma_{\mathrm{ext}}\mathrm{Im}\left(\sum_q\left|E_q\right|^2\partial_{\varphi}\Psi_q\right)=\frac{n_m}{Rkc}\sigma_{\mathrm{ext}}I(R)\Psi'(\varphi), \tag{6}$$

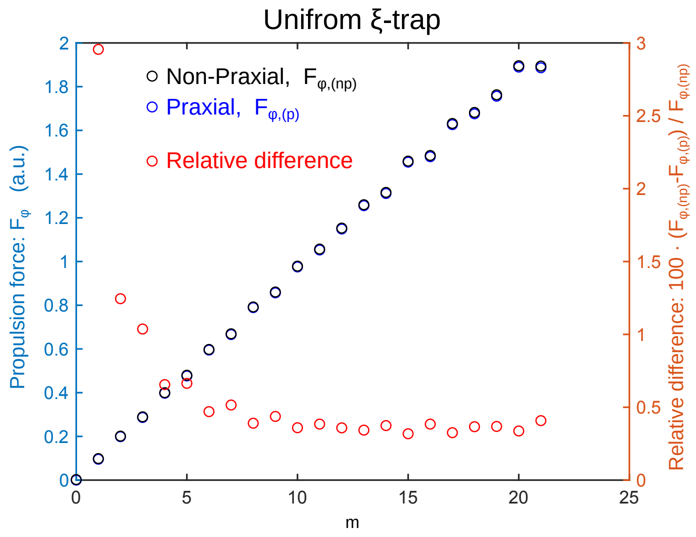

Figure S1. Graphical representation of the optical propulsion force magnitude (arbitrary units) corresponding to the paraxial and non-paraxial regime, displayed as a function of the order m (topological charge). The relative difference between these forces estimation is lower than 2 % in the considered interval of phase gradients.

where we have introduced the polar coordinates (r, φ) in the focal plane. For further analysis it is convenient to express the phase as

$$\Psi(\varphi) = m2\pi S\left(\varphi\right)/S(2\pi), \tag{7}$$

with $S(\varphi)$ being an arbitrary real function describing the phase distribution along the curve, while the parameter $m = \Psi(2\pi)/2\pi$ defines the global phase accumulation along the entire curve, which can be associated with beam generalized topological charge. The azimuthal component of scattering force tangential to the curve (circumference), further refereed to as optical propulsion force, is given by

$$F_\varphi(R,\varphi) = \frac{n_m \sigma_{\text{ext}}}{cRkS(2\pi)} mI(R)S'(\varphi) = \frac{\sigma_{\text{ext}}}{c} I(R)\xi(\varphi), \tag{8}$$

where $\xi(\varphi) = \Psi'(\varphi)/Rk_0 = mS'(\varphi)/Rk_0 S(2\pi)$ is a dimensionless function describing the strength of the phase gradient, see Eq. (6) and Eq. (7). The function $\xi(\varphi)$ is useful for proper comparison of the optical propulsion force between different trap configurations. In this study we have considered optical propulsion forces created by three different types of phase gradient profiles $\xi(\varphi)$. The corresponding phase $\Psi(\varphi)$ prescribed along the ring trap has been created with: $S(\varphi) = \varphi$ for the uniform ξ-trap, $S(\varphi) = 0.5\varphi + 0.35\Lambda(2\varphi)$ for the

2-sector $\xi_{1,2}$-trap (where $\Lambda(2\varphi)$ is the triangular wave function), and $S(\varphi) = \varphi + 0.5\varphi^2$ for the $\xi(\varphi)$-trap.

In the particular case of the uniform ξ-trap −its uniform phase distribution ($S(\varphi) = \varphi$) yields $\xi(\varphi) = m/Rk_0$− the associated polymorphic beam Eq. (4) is reduced to the following helical Bessel beam of order m:

$$\mathbf{E}_0\left(r,\phi\right) = \epsilon_{\pm} A_0 \frac{2\pi R^2}{\lambda \mathrm{f}} \exp\left[\mathrm{i}m\phi\right] J_m(kRr/\mathrm{f}), \tag{9}$$

and the corresponding optical propulsion force

$$F_{\varphi}(R,\varphi) = \frac{\sigma_{\mathrm{ext}}}{ck_0 R} m I(R), \tag{10}$$

is uniform along the whole curve.

When the helical Bessel beam is focused by a high numerical aperture objective lens, the optical propulsion force can be derived by applying the Eq. (2) and the Richards-Wolf expressions [13] further generalized for the circular polarized vortex beams [14, 15]. Thus we obtain

$$F_{\varphi}(R,\varphi) = \frac{\varepsilon_0 \varepsilon}{4} \sigma_{\mathrm{ext}} (kR)^3 A_0^2 \left[\left| m I_{0,m}(R) \right|^2 + (m \pm 2) \left| I_{2,m\pm2}(R) \right|^2 + 2(m \pm 1) \left| I_{1,m\pm1}(R) \right|^2 \right], \tag{11}$$

where:

$$\begin{aligned} I_{0,m}(r) &= \int_0^{\beta} J_m(kr\sin\theta) J_m(kR\sin\theta)(1+\cos\theta)\sin\theta\sqrt{\cos\theta}\exp\left(\mathrm{i}kz\cos\theta\right)\mathrm{d}\theta, \\ I_{2,m\pm2}(r) &= \int_0^{\beta} J_{m\pm2}(kr\sin\theta) J_m(kR\sin\theta)(1-\cos\theta)\sin\theta\sqrt{\cos\theta}\exp\left(\mathrm{i}kz\cos\theta\right)\mathrm{d}\theta, \\ I_{1,m\pm1}(r) &= \int_0^{\beta} J_{m\pm1}(kr\sin\theta) J_m(kR\sin\theta)\sin^2\theta\sqrt{\cos\theta}\exp\left(\mathrm{i}kz\cos\theta\right)\mathrm{d}\theta, \end{aligned} \tag{12}$$

with $\beta = \arcsin(\mathrm{NA}/n_{\mathrm{imm}})$ and NA being the numerical aperture of the objective, where n_{imm} is the refractive index of immersion medium, whereas symbols $\pm$ correspond to the circular polarization vector $\epsilon_{\pm} = (1, \pm\mathrm{i})$. The irradiance is given by

$$I(R) = \frac{n_m \varepsilon_0 c}{4} (kR)^4 A_0^2 \left[\left| I_{0,m}(R) \right|^2 + \left| I_{2,m\pm2}(R) \right|^2 + 2\left| I_{1,m\pm1}(R) \right|^2 \right]. \tag{13}$$

As we observe from the Fig. S1 there is no significant difference between the values of the azimuthal propulsion force calculated from the Eq. (11) and from the expression Eq. (10)

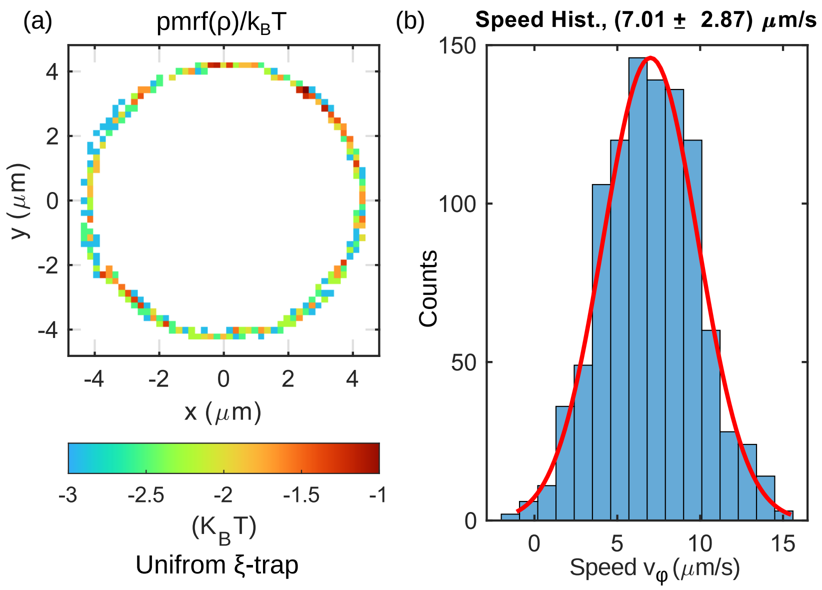

Figure S2. (a) Measured potential of mean confinement radial-force (pmrf), which represents an effective radial trapping potential in the uniform ξ-trap. The data confirms the stable confinement of a single (resonant) NP in the uniform ξ-trap. (b) Tangential speed (v_φ) histogram of the NP optically transported in the uniform ξ-trap. The NP has a mean speed of $\langle v_\varphi \rangle = 7\,\mu\text{m/s}$ and speed standard deviation of $2.87\,\mu\text{m/s}$. The red curve corresponds to the Gaussian fit.

obtained in paraxial approximation. The following parameters have been used in the simulations: $\epsilon_+ = (1, +\mathrm{i})$, $\beta = 0.95$, $\lambda_0 = 532\mathrm{nm}$, and $R = 4\mu\mathrm{m}$. We also observe that in the considered interval of the vortex topological charge $m \in [0, 20]$, which corresponds to the phase gradient interval of freestyle optical trap applied in our experiments, the force is increased linearly with m. We underline this fact since, in general, the finite aperture of the objective makes difficult the focusing of high order Bessel beams in a "perfect" vortex ring.

The azimuthal (propulsion) force calculated using Eq. (6) has been compared with the one obtained by using numerical methods based on the generalized Lorenz-Mie theory (GLMT) that allow for more accurate calculation of the forces beyond the Rayleigh approximation. By using the so-called optical tweezers GLMT toolbox reported in Ref. [16] we have calculated the total force exerted on a spherical gold NP with radius $a = 200$ nm, immersed in water ($n_m = 1.34$) by a circular polarized vortex Bessel beam ($\lambda_0 = 532$ nm and $m = -20$) focused by a high-numerical-aperture objective lens (1.4 NA, $100\times$). The magnitude of the propulsion force obtained by this method differs in about 10 % from the one given by Eq. (6). Therefore,

it can be assumed that Eq. (6) is valid for the estimation of propulsion force in the considered experimental conditions. In particular, the propulsion force Eq. (6) has been used in the numerical simulations of the NP motion (based on the 2D Langevin dynamics equation) in the three considered ring traps, as described in the next section.

The position tracking of the NP has been performed by using an open source software [17]. Using the probability density function $P(\rho)$ from the radial ($\rho = R - \sqrt{x^2 + y^2}$) position histograms, we have calculated the potential of mean confinement radial-force (pmrf), which represents an effective radial trapping potential: $\mathrm{pmrf}(\rho) = -k_B T \log P(\rho)$, where k_B is the Boltzmann's constant and T the temperature of thermal bath. The radial positional stiffness of the trap can be estimated as $k_r = k_B T / \langle \rho^2 \rangle$, with $\langle \rho^2 \rangle$ being the square of the position standard deviation. In Fig. S2(a) the $\mathrm{pmrf}(\rho)$ distribution is displayed confirming a reasonably good radial confinement (a mean potential value of $-2.6 k_B T$) of the NP in the uniform ξ-trap, with an estimated radial stiffness $k_r = 1.3\,\mathrm{pN}/\mu\mathrm{m}$. Similar k_r values have been obtained in the rest of the studied ring traps. The velocity histogram of the NP confined in the uniform ξ-trap, displayed in Fig. S2(b), fits well to a normal distribution of speed (red line) with mean speed $\langle v_\varphi \rangle = 7\,\mu\mathrm{m/s}$ and speed standard deviation of $2.87\,\mu\mathrm{m/s}$. The Brownian thermal force is indeed the origin of the NP position fluctuations yielding the observed distribution of velocities, as expected.

SIMULATION METHOD OF 2D LIGHT-DRIVEN MOTION OF COLLOIDAL PARTICLE

The transverse optical propulsion force $\mathbf{F} = F_\varphi(R, \varphi)\mathbf{u}_\varphi$, Eq. (8) with $\mathbf{u}_\varphi$ being the unit vector tangent to the curve, acting upon the NP induces a dynamics described by the 2D Langevin equation of motion

$$M\ddot{\mathbf{r}} = \mathbf{F} - \nu\dot{\mathbf{r}} + \zeta(t), \tag{14}$$

where $\mathbf{r} = \mathbf{r}(t) = (x_{NP}(t), y_{NP}(t))$ is the position of the NP of mass M, $\mathbf{v} = \dot{\mathbf{r}} = \mathrm{d}\mathbf{r}/\mathrm{d}t$ is the speed of the particle, ν is the Stokes drag friction coefficient, and $\zeta(t)$ is the stochastic thermal noise term responsible for Brownian motion of the particle [18–21]. The noise term $\zeta(t)$ follows a Gaussian probability distribution such that $\langle \zeta_p(t)\zeta_q(t') \rangle = 2\nu k_B T \delta_{p,q}\delta(t - t')$, where: the angle brackets denote an average over time, $\delta_{p,q}$ is a Kronecker delta function over the coordinate indices and $\delta(t - t')$ is a delta function of time. The drag friction coefficient is given by the expression $\nu = 6\pi a \eta$ where η is the dynamic viscosity of the medium. Since the

considered spherical particle is transported near the glass cover-slip there exists an increase in the hydrodynamic drag approximated by the expression [22]

$$\nu = \frac{6\pi a\eta}{1 - \frac{9}{16}\left(\frac{a}{h}\right) + \frac{1}{8}\left(\frac{a}{h}\right)^3 - \frac{45}{256}\left(\frac{a}{h}\right)^4 - \frac{1}{16}\left(\frac{a}{h}\right)^5}, \tag{15}$$

that depends on the particle radius a and the distance h between the particle and the substrate (glass cover-slip) where the particle is trapped.

The equation of motion Eq. (14) can be solved by using a splitting-method time-integration scheme (BAOAB) [18–20]: Eq. (14) is split into three parts labelled A, B, and O corresponding to the kinetic term, potential term, and friction-thermal fluctuation term, respectively. In the BAOAB method the integration sequence is [18]:

$$\begin{aligned}
\mathbf{v}^{(j+1/2)} &= \mathbf{v}^{(j)} + \mathbf{F}^{(j)}\frac{\Delta t}{2M}, \ (B) \\
\mathbf{r}^{(j+1/2)} &= \mathbf{r}^{(j)} + \mathbf{v}^{(j+1/2)}\frac{\Delta t}{2}, \ (A) \\
\hat{\mathbf{v}}^{(j+1/2)} &= c_1\mathbf{v}^{(j+1/2)} + \beta^{(j+1)}\frac{c_3}{\sqrt{M}}, \ (O) \\
\mathbf{r}^{(j+1)} &= \mathbf{r}^{(j+1/2)} + \hat{\mathbf{v}}^{(j+1/2)}\frac{\Delta t}{2}, \ (A) \\
\mathbf{v}^{(j+1)} &= \hat{\mathbf{v}}^{(j+1/2)} + \mathbf{F}^{(j+1)}\frac{\Delta t}{2M}, \ (B)
\end{aligned} \tag{16}$$

where the index j denotes the temporal dependence as $\mathbf{r}^{(j)} = \mathbf{r}(t)$, thus $\mathbf{r}^{(j+1)} = \mathbf{r}(t + \Delta t)$, while $\mathbf{F}^{(j)} = \mathbf{F}(\mathbf{r}(t))$. The friction-thermal fluctuation term has the following parameters: $c_1 = \exp(-\gamma\Delta t)$, $c_2 = (1 - c_1)/\gamma$ and $c_3 = \sqrt{k_B T(1 - c_1^2)}$ with $\gamma = \nu/M$ and $\beta^{(j)}$ being a white noise function (a set of normally distributed random numbers, ranging from 0 to 1, thus with mean 0 and variance 1). Note that such a noise contribution can be very small if $|\mathbf{F}| > |\zeta(t)|$. The integration problem for the non-conservative force field $\mathbf{F}$ is studied in detail in [20].

In our case the medium is water and the NP is strongly confined by the laser curve against the glass cover-slip such that $h \sim a$ can be assumed, see [12]. Therefore, the particle trajectory can be well approximated by the 2D curve's shape $\mathbf{R}(\varphi) = (R\cos\varphi, R\sin\varphi)$. All these facts allow the simplification of Eq. (16) to a 1-dimensional equation motion along the curve $\mathbf{R}(\varphi)$. Indeed, the set of expressions Eq. (16) provide the velocity $\mathbf{v}(\varphi(t))$ and position $\mathbf{r}(t) = \mathbf{R}(\varphi(t))$ of the particle confined in the curve. In the experimental results the NP experiences a strong radial confinement in the ring trap such $|R(\varphi) - R| \leq 100$ nm, which supports the assumed 1-dimensional NP motion along the curve. In the simulation of the

light-driven particle motion we have considered a very small time-step value $\Delta t = 0.1$ ms, which is a requirement when the force field $\mathbf{F}$ is stiff [18–20].

In practice, it is often assumed that the motion of the NP is over-damped, $\ddot{\mathbf{r}} = 0$, thus the equation of motion could be simplified as

$$\dot{\mathbf{r}} = \mathbf{F}(\mathbf{R}(\varphi(t)))/\nu = \mathbf{v}(\varphi(t)), \tag{17}$$

that in turn allows for the estimation of the force field $\mathbf{F}$ (propulsion force) from the measurement of the speed obtained from the particle tracking data.

TEMPERATURE INCREASE OF METAL NPS AND SURROUNDING LIQUID

The temperature increase ΔT_{NP} experienced by a single spherical NP embedded in a homogenous medium (e.g., a fluid) of thermal conductivity κ is given by the expression [23]

$$\Delta T_{NP} = \frac{I\sigma_{\text{abs}}}{4\pi\kappa a}. \tag{18}$$

When the NP is located close to the substrate (glass) the thermal conductivity is given as an average $\kappa = (\kappa_w + \kappa_s)/2$, where $\kappa_w = 0.6$ W/(m·K) and $\kappa_s = 1.38$ W/(m·K) is the thermal conductivity of water and glass, correspondingly, considered in our case. The absorption cross section $\sigma_{\text{abs}} = 0.137\,\mu\text{m}^2$ of the considered gold NP (radius $a = 200$ nm) has been calculated applying the Mie theory. In the experiment (e.g., in the case of the $\xi(\varphi)$-trap) we have used an irradiance $I = 0.73\,\text{mW}/\mu\text{m}^2$ and therefore $\Delta T_{NP} \sim 40$ K. The uniform-temperature approximation inside the metal NP can be assumed [24].

Outside the hot NP the steady-state (equilibrium) distribution of temperature increase of the surrounding water can be calculated by using the expression $\Delta T(r) = \Delta T_{NP} a/r$ [24], see Fig. S3(a), where $r > a$ is the radial distance from the centre of the particle. Since thermal processes are much faster in the metal, the surrounding fluid (water) governs the value of the time scale to reach the steady-state regime, which according to the study carried out in [24] is $t = a^2/D_{water}$ with $D_{water} = 1.43 \cdot 10^{-8}\,\text{m}^2/\text{s}$ being the thermal diffusivity of water. In our case $t \sim 2.8\,\mu\text{s}$ and therefore the equilibrium distribution of temperature increase $\Delta T(r)$ is reached almost instantaneously as the NP moves (driven by any phase gradient) around the ring trap. Moreover, due to the low Rayleigh number of water, the thermal-induced fluid convection is not supposed to distort the temperature distribution within the liquid, regardless

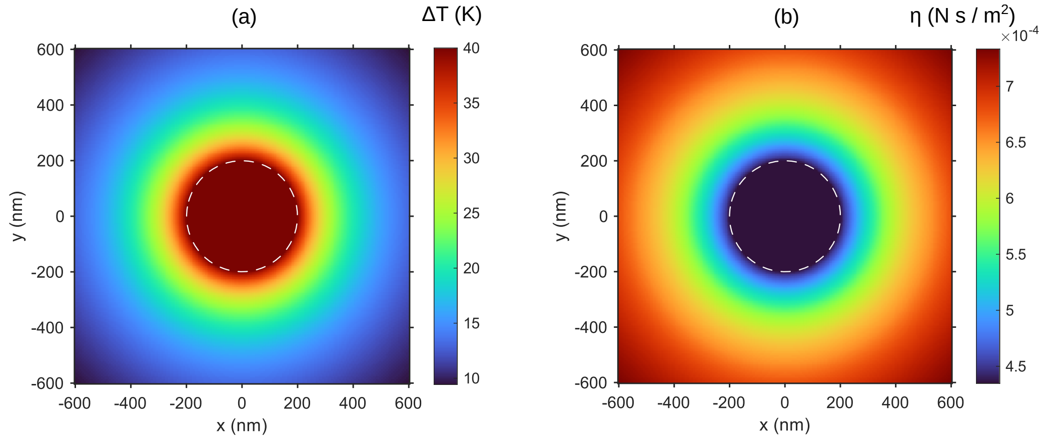

Figure S3. (a) Equilibrium distribution of temperature increase $\Delta T(r)$ of the water surrounding the hot NP (gold nano-sphere of radius $a = 200$ nm), indicated by the white dashed-circle. (b) Distribution of the dynamic viscosity $\eta(T + \Delta T(r))$ corresponding to the water surrounding the hot NP, where $T = 298$ K.

of the temperature increase or the size of the heat source [25]. Then the spatial distribution of temperature around the moving NP is the same as around a static one.

The dynamic viscosity of the water surrounding the NP varies as a function of the temperature [26] and therefore of the position as $\eta(298\,\mathrm{K} + \Delta T(r))$. In Fig. S3(b) the spatial distribution of $\eta(298\,\mathrm{K}+\Delta T(r))$ around the considered NP is displayed. The most rapid NP moves with a speed of $20\,\mu\mathrm{m/s}$ therefore in a time $\sim 2.8\,\mu\mathrm{s}$ (required to reach the temperature steady-state regime) the particle only travels a distance of 0.056 nm. Thus, the temperature rise of the surrounding water at this distance can be approximated as $\Delta T_{NP} = 40$ K, see Fig. S3(a). Then, one can estimate the dynamic viscosity value of the water surrounding the NP as $\eta(298\,\mathrm{K} + \Delta T_{NP}) = 4.5 \cdot 10^{-4}\,\mathrm{Ns/m^2}$. This value of η has been used in the numerical simulation of the NP motion driven by the optical propulsion force, which is in good agreement with the experimental results as explained in the main text.

As it has been mentioned in the main text, the local increase of the temperature in the interval $\Delta T \sim 70 - 250\,\mathrm{K}$ produces a convective fluid flow arising from the temperature-induced Marangoni effect at the liquid water/superheated water interface due to nanobubbles formation [27]. This fluid flow could explain the motion of tracer NPs toward the G-NP (heat source) observed in our experiments. Since the G-NP is a dynamic assembly of NPs (identical nano-spheres) with varying number of NPs and inter-particle distances an exact calculation

of its temperature rise is challenging. Thus, in order to estimate the temperature rise, we simplify the problem considering only the NPs that comprise the G-NP core. They are in close proximity but not attached to each other, in part, because the NPs are coated with charged surfactants that prevent from particle contact and aggregation. It is supported by the fact that NP clustering has not been observed in our experiments. The temperature of the G-NP could be estimated following the expressions given in [23], however, in such a case all the inter-particle distances have to be known. Alternatively, if the inter-particle distances are unknown, the temperature rise ΔT_S at the centre of a structure comprising such NPs can be estimated by using the expression derived in [28] for dispersed NPs in a fluid

$$\Delta T_S = \rho_N a_S^2 I \sigma_{\mathrm{abs}}/2\kappa = 2\pi a_S^2 a \rho_N \Delta T_{NP}, \tag{19}$$

where a_S is the radius of the heated region, ρ_N is the concentration of hot NPs. In our case, this heated structure corresponds to the core of the G-NP comprising N NPs of radius $a = 200$ nm grouped in form of disk with radius a_S and heigh $2a$. Thus, the concentration in the core region of the G-NP is $\rho_N = N/V$ with $V = 2\pi a_s^2 a$ being the disk volume. Therefore, the temperature increase ΔT_{GNP} at the centre of the G-NP (at its core) can be estimated from Eq. (19) as it follows

$$\Delta T_{GNP} = N \Delta T_{NP}, \tag{20}$$

which is consistent with the fact of superposition of temperature fields from all the hot NPs. This collective thermal behaviour can create a homogenous heat source distributed throughout the entire core region of the G-NP [28]. The measured average radius of the disk-like core of the G-NP is $a_S \sim 0.6\,\mu\mathrm{m}$, which gives a number of $N \sim 5$ hot NPs comprising the core. Thus, the G-NP transported in the $\xi(\varphi)$-trap (with $I = 0.73\,\mathrm{mW}/\mu\mathrm{m}^2$) experiences a temperature rise $\Delta T_{GNP} \sim 200$ K. Note that the plasmonic coupling between the NPs leads to a red shift of the plasmonic resonance and therefore the temperature rise (ΔT_{NP}) of a NP can be lower in the core than in the case of a single one. However, even if the ΔT_{NP} value is decreased by a factor of two the resulting estimated temperature rise of the G-NP, 100 K, is compatible with appearance of nanobubbles formation [27, 29]. On the other hand, the estimated temperature of the superheated water $400 - 500$ K surrounding the G-NP is less than the reported threshold for microbubbles formation (580 ± 20 K) [30, 31].

In the experiments we have also observed that some NPs are eventually expelled from the G-NP's core when there are more than $N \sim 6$ particles. This could be explained by

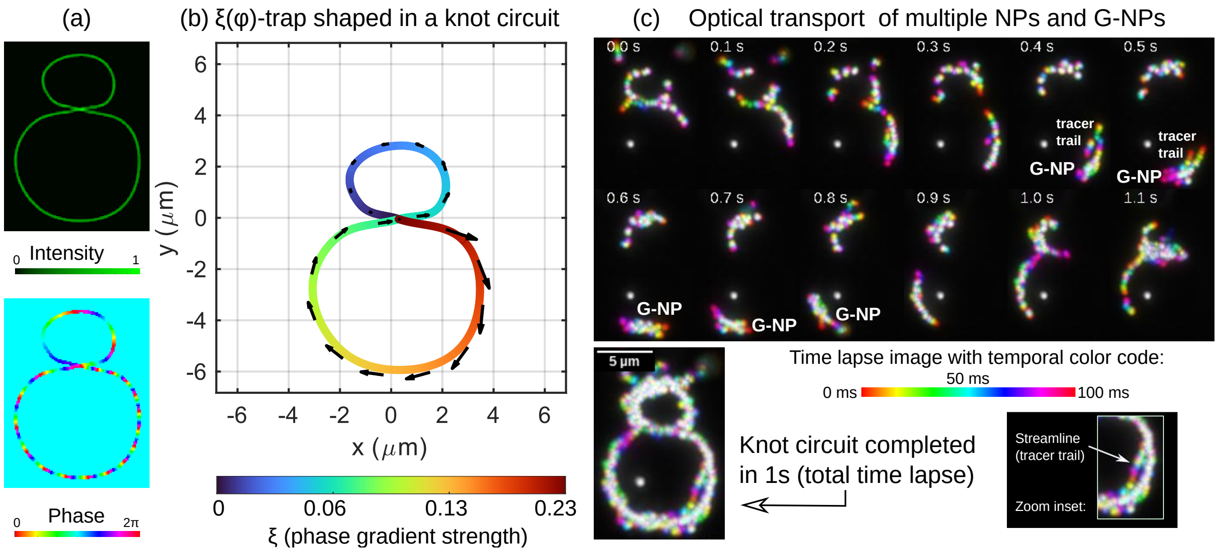

Figure S4. (a) Intensity and phase distributions of a $\xi(\varphi)$-trap shaped in form of knot circuit. (b) Phase gradient strength prescribed in the knot circuit. (c) Multiple resonant NPs are transported in the knot-circuit $\xi(\varphi)$-trap driven by the phase-gradient force. The time lapse image series demonstrate the confinement and transport of the NPs as well as the creation of G-NPs, see Supplementary video S7. The NPs travel around the whole knot circuit as the total time lapse image reveals (merged time series image captured in 1 s).

an increased value of the core's temperature rise (i.e., $\Delta T_{GNP} \sim 250$ K) that leads to a temperature of the superheated water near the threshold for microbubbles formation, thereby creating a stronger water flow able to expel NPs from the core. We think this could be an evidence of a temperature rise limit for the formation of a stable G-NP core. Taking into account the temperature rise limit $\sim 250\,\mathrm{K}$ for the nanobubbles regime we infer that a threshold number of NPs comprising a stable core could be $N \sim 250\,\mathrm{K}/\Delta T_{NP}$, which coincides with $N \sim 6$ for $\Delta T_{NP} \sim 40$ K. Nevertheless, a further study is required to completely confirm this hypothesis.

OPTICAL TRANSPORT ALONG ARBITRARY TRAJECTORIES

In this work we have applied a freestyle trap [8, 9] whose shape and propulsion force can be easily adapted to the standing application. As an example, in the main text, we have

studied a ring trap with tailored phase-gradient propulsion force to optically transport single and multiple resonant NPs. In this section, we demonstrate that indeed the shape of the optical trap can be arbitrary while preserving the independent control of the $\xi(\varphi)$-tailored optical propulsion force. In particular, we have considered a knot (eight-shaped) circuit [32] for the optical transport of the gold NPs as shown in Fig. S4. Specifically, the intensity and phase distribution of the trapping beam are displayed in Fig. S4(a), where the phase gradient prescribed along the circuit corresponds to the linear increasing $\xi(\varphi)$-trap as shown in Fig. S4(b). The corresponding phase-gradient propulsion force drive the transport of the NPs around the whole circuit performing several loops (see Supplementary video S7), as an example, in Fig. S4(c) it has been shown one loop performed in a time of ~ 1 s. As the NPs travel around the circuit their speed change and several G-NPs can be created as observed in Fig. S4(c) and Supplementary video S7. Multiple NPs and G-NPs were transported in the knot circuit whose shape is revealed by the NPs trails observed in the time lapse images, see Fig. S4(c). The trail of the tracer NP reveals the streamline of the convective water flow created by the G-NPs, as in the case of ring traps. This example confirms the ability to optically transport NPs and G-NP optothermal convertors along complex trajectories. Note that this experiment has been also recorded at 100 frames per second, the time lapse image series has been created from the raw data by using a time lapse interval of 100 ms. Further information about the generation of the applied freestyle laser traps for programmable optical transport can be found in [9, 32].

[1] Chaumet, P. C. & Nieto-Vesperinas, M. Time-averaged total force on a dipolar sphere in an electromagnetic field. *Optics Letters* **25**, 1065 (2000). 0305043.

[2] Gao, D. *et al.* Optical manipulation from the microscale to the nanoscale: Fundamentals, advances and prospects. *Light: Science and Applications* **6**, e17039–e17039 (2017).

[3] Zemánek, P., Volpe, G., Jonáš, A. & Brzobohatý, O. Perspective on light-induced transport of particles: from optical forces to phoretic motion. *Advances in Optics and Photonics* **11**, 577 (2019).

[4] Albaladejo, S., Marques, M. I., Laroche, M. & Saenz, J. J. Scattering forces from the curl of the spin angular momentum of a light field. *Physical Review Letters* **102**, 1–4 (2009).

[5] Ruffner, D. B. & Grier, D. G. Comment on: Scattering Forces from the Curl of the Spin Angular Momentum of a Light Field. *Physical Review Letters* **111**, 059301 (2013).

[6] Rodrigo, J. A. & Alieva, T. Light-driven transport of plasmonic nanoparticles on demand. *Sci. Rep.* **6** (2016).

[7] Bohren, C. F. & Huffman, D. R. Absorption and Scattering by a Sphere. *Absorption and Scattering of Light by Small Particles* 82–129 (2007).

[8] Rodrigo, J. A. & Alieva, T. Freestyle 3D laser traps: tools for studying light-driven particle dynamics and beyond. *Optica* **2**, 812 (2015).

[9] Rodrigo, J. A., Angulo, M. & Alieva, T. Dynamic morphing of 3D curved laser traps for all-optical manipulation of particles. *Opt. Express* **26**, 18608 (2018).

[10] Rodrigo, J. A. & Alieva, T. Polymorphic beams and Nature inspired circuits for optical current. *Sci. Rep.* **6** (2016).

[11] Lehmuskero, A., Li, Y., Johansson, P. & Käll, M. Plasmonic particles set into fast orbital motion by an optical vortex beam. *Optics Express* **22**, 4349 (2014).

[12] Figliozzi, P. *et al.* Driven optical matter: Dynamics of electrodynamically coupled nanoparticles in an optical ring vortex. *Physical Review E* **95**, 022604 (2017).

[13] Richards, B. & Wolf, E. Electromagnetic diffraction in optical systems, ii. structure of the image field in an aplanatic system. *Proceedings of the Royal Society of London. Series A. Mathematical and Physical Sciences* **253**, 358–379 (1959).

[14] Chen, B. & Pu, J. Tight focusing of elliptically polarized vortex beams. *Appl. Opt.* **48**, 1288–1294 (2009).

[15] Kotlyar, V. V., Nalimov, A. G. & Stafeev, S. S. Energy backflow in the focus of an optical vortex. *Laser Physics* **28**, 126203 (2018).

[16] Nieminen, T. A. *et al.* Optical tweezers computational toolbox. *Journal of Optics A: Pure and Applied Optics* **9** (2007).

[17] Tinevez, J.-Y. *et al.* Trackmate: An open and extensible platform for single-particle tracking. *Methods* **115**, 80 – 90 (2017).

[18] Leimkuhler, B. & Matthews, C. Rational construction of stochastic numerical methods for molecular sampling. *Applied Mathematics Research eXpress* **2013**, 34–56 (2013). 1203.5428.

[19] Sule, N., Rice, S. A., Gray, S. K. & Scherer, N. F. An electrodynamics-Langevin dynamics (ED-LD) approach to simulate metal nanoparticle interactions and motion. *Optics Express* **23**, 29978 (2015).

[20] Sachs, M., Leimkuhler, B. & Danos, V. Langevin dynamics with variable coefficients and nonconservative forces: From stationary states to numerical methods. *Entropy* **19** (2017).

[21] Shi, Y. *et al.* Nanometer-precision linear sorting with synchronized optofluidic dual barriers. *Science Advances* **4** (2018). https://advances.sciencemag.org/content/4/1/eaao0773.full.pdf.

[22] Neuman, K. C. & Block, S. M. Optical trapping. *Review of Scientific Instruments* **75**, 2787–2809 (2004).

[23] Baffou, G. *et al.* Photoinduced heating of nanoparticle arrays. *ACS Nano* **7**, 6478–6488 (2013).

[24] Baffou, G., Quidant, R. & García De Abajo, F. J. Nanoscale control of optical heating in complex plasmonic systems. In *ACS Nano*, vol. 4, 709–716 (2010).

[25] Donner, J. S., Baffou, G., McCloskey, D. & Quidant, R. Plasmon-assisted optofluidics. *ACS Nano* **5**, 5457–5462 (2011).

[26] Peterman, E. J., Gittes, F. & Schmidt, C. F. Laser-induced heating in optical traps. *Biophysical Journal* **84**, 1308–1316 (2003).

[27] Chikazawa, J. I., Uwada, T., Furube, A. & Hashimoto, S. Flow-induced transport via optical heating of a single gold nanoparticle. *Journal of Physical Chemistry C* **123**, 4512–4522 (2019).

[28] Keblinski, P., Cahill, D. G., Bodapati, A., Sullivan, C. R. & Taton, T. A. Limits of localized heating by electromagnetically excited nanoparticles. *Journal of Applied Physics* **100**, 1–6 (2006).

[29] Hou, L., Yorulmaz, M., Verhart, N. R. & Orrit, M. Explosive formation and dynamics of vapor nanobubbles around a continuously heated gold nanosphere. *New Journal of Physics* **17** (2015).

[30] Baffou, G., Polleux, J., Rigneault, H. & Monneret, S. Super-heating and micro-bubble generation around plasmonic nanoparticles under cw illumination. *Journal of Physical Chemistry C* **118**, 4890–4898 (2014).

[31] Baral, S., Green, A. J., Livshits, M. Y., Govorov, A. O. & Richardson, H. H. Comparison of vapor formation of water at the solid/water interface to colloidal solutions using optically excited gold nanostructures. *ACS Nano* **8**, 1439–1448 (2014).

[32] Rodrigo, J. A., Angulo, M. & Alieva, T. Programmable optical transport of particles in knot circuits and networks. *Opt. Lett.* **43**, 4244 (2018).
